# Primary healthcare provider perspectives on hypertensive disorders of pregnancy in southwestern Uganda: a qualitative study

**DOI:** 10.1186/s12913-026-15315-2

**Published:** 2026-08-06

**Authors:** Henry Mark Lugobe, Josephine Nambi Najjuma, Ian B. Wilkinson, David Collins Agaba, Leevan Tibaijuka, Alan Babweteera, Wasswa George Muganwa, Simon Tomusange, Kwame Adu-Bonsaffoh, Pius Musinguzi, Gerald Mwebembezi, Jonathan Izudi, Carmel M. Mceniery

**Affiliations:** 1 Department of Obstetrics and Gynecology, Mbarara University of Science and Technology, Mbarara, Uganda; 2 Division of Experimental Medicine and Immunotherapeutics, Department of Medicine, University of Cambridge, Cambridge, UK; 3 Department of Nursing, Mbarara University of Science and Technology, Mbarara, Uganda; 4 Department of Physiology, Mbarara University of Science and Technology, Mbarara, Uganda; 5 Department of Obstetrics and Gynecology, Mbarara Regional Referral Hospital, Mbarara, Uganda; 6 Department of Obstetrics and Gynecology, Kawempe National Referral Hospital, Kampala, Uganda; 7 Department of Obstetrics and Gynecology, University of Ghana medical school, Accra, Ghana; 8 Directorate of Graduate Training, Research and Innovations, Muni University, Arua, Uganda; 9 Department of Community Health, Mbarara University of Science and Technology, Mbarara, Uganda

**Keywords:** Hypertensive disorders of pregnancy, Screening, Diagnosis, Management, Healthcare providers, Uganda

## Abstract

**Background:**

Hypertensive disorders of pregnancy (HDP), specifically preeclampsia and eclampsia, are leading causes of maternal and perinatal mortality in sub-Saharan Africa. In Uganda’s tiered health system, primary healthcare facilities (Health Center III and IV) serve as the frontline for early detection of HDP among women attending antenatal care (ANC). However, implementation gaps persist in these settings. This study explored the perspectives of healthcare providers (HCPs) on the practices, barriers, and facilitators regarding HDP screening, diagnosis, and management in Southwestern Uganda.

**Methods:**

We conducted an exploratory qualitative study using in-depth interviews with 13 purposively sampled HCPs (midwives, nurses, and medical officers) across six primary health facilities in Mbarara City. Using a semi-structured interview guide, data were collected from January 2025 to March 2025. Data were analyzed using inductive thematic content analysis.

**Results:**

Screening for HDP during ANC was primarily conducted using blood pressure measurement and urinalysis, but was frequently undermined by technical barriers such as non-functional blood pressure machines and supply constraints like stock-outs of urine dipsticks. No participants (0/13) reported the use of prophylactic aspirin for high-risk mothers. Key barriers included the absence of HDP-specific guidelines, heavy workloads, cultural and socioeconomic factors. Facilitators included routine health education sessions and the use of digital consultation via WhatsApp groups for real-time specialist support. To address these challenges, HCPs proposed potential measures such as facility-based mentorship for HCPs, the integration of mHealth decision-support tools, and redesigning ANC cards to make risk factors more visible.

**Conclusions:**

Healthcare providers in southwestern Uganda showed strong awareness of HDP care but were constrained by unreliable equipment, supply shortages, and limited staffing. Strengthening supply chains, mentorship, and digital decision-support could enhance the capacity of primary care facilities to deliver timely HDP prevention, screening, and early diagnosis.

**Supplementary Information:**

The online version contains supplementary material available at https://doi.org/10.1186/s12913-026-15315-2.

## Background

Hypertensive disorders of pregnancy (HDP), specifically preeclampsia and eclampsia, affect approximately 10% of pregnancies in sub-Saharan Africa [1], and remain a leading cause of maternal and perinatal morbidity and mortality. In southwestern Uganda, the clinical burden is particularly high, with a significant majority of patients presenting at tertiary referral centers already exhibiting severe features or eclampsia [2]. While the long-term cardiovascular and obstetric risks of HDP are well-documented [3, 4], global health priorities have shifted toward early identification and prevention through risk-factor screening, routine blood pressure monitoring, and the use of prophylactic aspirin [5]. However, challenges persist, especially in settings where health systems face constraints in infrastructure and human resources [6].

In the Ugandan health system, primary health care facilities (Health Center IIIs and IVs) serve as the essential first point of contact for pregnant women. These facilities are the critical frontline for HDP screening; Health Center IIIs (HCIII) provide Basic Emergency Obstetric Care (BEmONC) led by midwives, while Health Center IVs (HCIV) offer Comprehensive Emergency Obstetric Care (CEmONC) including surgical capacity [7]. Despite the strategic importance of these levels, a significant implementation gap persists. Evidence suggests that women referred from these primary centers to regional hospitals often arrive with advanced, life-threatening complications, indicating that the window for early screening and stabilization is being missed [2, 8].

While existing literature has extensively described the clinical outcomes of HDP at tertiary hospitals [2], there is a paucity of evidence exploring the process of care at the primary level. Specifically, we lack an in-depth understanding of how frontline healthcare providers (HCPs) navigate systemic constraints to implement HDP protocols.

Understanding the barriers and facilitators encountered by midwives and medical officers at the lower level health facilities is essential. Without this perspective, interventions designed at the policy level risk being culturally or logistically incompatible with the realities of primary care. Therefore, this study sought to explore the perspectives of HCPs at HCIII and HCIV facilities in southwestern Uganda regarding the screening, diagnosis, and management of HDP. These findings aim to inform the development of context-specific interventions that strengthen the primary health care response to HDP.

## Methods

### Study design and setting

This was an exploratory qualitative study using an inductive approach to identify the perspectives of healthcare providers (HCPs) on the screening, diagnosis, and management of HDP. The study was conducted between January 2025 and March 2025 across all six primary public health facilities in Mbarara City, Southwestern Uganda (five HCIIIs and one HCIV). The facilities were; Mbarara City HCIV which has 3 doctors and 14 midwives with ANC average attendance of 1664 mothers monthly and 100 deliveries per month. Nyamitanga HCIII has 3 midwives with ANC average attendance of 290 mothers monthly and 20 deliveries per month. Kakoba HCIII has 6 midwives with ANC average attendance of 400 mothers monthly and 40 deliveries per month. Biharwe HC III has 2 midwives with ANC average attendance of 300 mothers monthly and 35 deliveries per month. Nyakayojo HCIII has 2 midwives with ANC average attendance of 430 mothers monthly and 50 deliveries per month. Kyarwabuganda HCIII has 2 midwives with ANC average attendance of 237 mothers monthly and 20 deliveries per month.

According to the Ugandan health system, HCIIIs serve a sub-county catchment of approximately 20,000 people and the maternity services are primarily midwife-led, providing Basic Emergency Obstetric Care (BEmONC). HCIVs serve approximately 100,000 people and provide Comprehensive Emergency Obstetric Care (CEmONC), including cesarean sections, and are staffed by at least one Medical Officer [9]. Taken together, these facilities represent the primary level of care where the majority of pregnant women in Uganda first seek antenatal services. Health center III have limited to no laboratory and ultrasound services while Health center IVs have both laboratory and ultrasound services.

### Research team and reflexivity

The study was conducted and reported in accordance with the Consolidated Criteria for Reporting Qualitative Research (COREQ). Data collection was performed by two research assistants (GM and PM), who are nurses trained in qualitative interviewing with prior experience in health systems research. Prior to obtaining written informed consent, participants were informed that the study’s purpose was to identify gaps in HDP care to inform future clinical interventions. The researchers had no prior personal or professional interaction with the study participants. To address potential researcher bias and power dynamics, the team engaged in active reflexive practice throughout the study design, data collection, and analysis. Data collection was conducted by nurses interviewing predominantly midwives, power asymmetries related to clinical hierarchy were minimized since the interviewers held similar or lower clinical positions than some respondents. However, to mitigate potential social desirability bias regarding routine clinical practices, interviewers maintained a non-judgmental, peer-to-peer approach and conducted interviews in a setting that ensured privacy. The broader research team brought complementary perspectives. HML, a consultant obstetrician, provided expertise in clinical preeclampsia management, while JN, a senior nurse researcher, provided qualitative methodological expertise. To prevent clinical assumptions from driving data interpretation, the analysis of the data was led independently by PM and GM. Any discrepancies and emerging themes were critically examined during team consensus meetings involving HML and JN, ensuring that data interpretation remained grounded in the participants’ voices rather than predetermined clinical expectations.

### Study participants and sampling

We used purposive sampling to recruit a diverse group of healthcare providers (HCPs), ensuring a representation of various clinical roles across all six study sites. Eligible participants included medical officers, clinical officers, nursing officers, and midwives directly involved in providing antenatal care (ANC). We purposively selected at least one HCP from each of the health facilities until thematic saturation was achieved.

Participants were required to have at least six months of experience at their current facility. This duration was chosen to ensure that the HCPs had sufficient longitudinal exposure to the facility’s specific supply chain cycles, patient volume trends, and referral logistics, thereby providing information-rich data regarding HDP care at the primary level.

The distribution of cadres in our sample with predominantly midwives (*n* = 8), clinical officers (*n* = 3), nurses (*n* = 1) and one medical officer (*n* = 1) reflects the structural staff composition of primary care facilities in Uganda. Health Center IIIs are midwife led facilities providing Basic Emergency Obstetric Care (BEmONC) without resident medical officers (doctors), whereas Health Center IVs offer Comprehensive Emergency Obstetric Care (CEmONC) and are staffed by medical officers. Therefore, our sample captures the frontline cadre distribution responsible for routine ANC screening and triage in this setting.

### Data collection

Data were collected using a semi-structured in-depth interview guide (Supplementary file 1). Interviews were conducted in English and in private rooms within the health facilities to ensure confidentiality. Each in-depth interview lasted 45 to 60 min. Data collection continued until thematic saturation was reached—the point where no new themes or sub-themes emerged from subsequent interviews [10]. Saturation was achieved after 13 interviews. All sessions were audio-recorded, and field notes were maintained to document non-verbal cues and contextual factors. The interview guide was structured around: current practices, barriers, facilitators, and potential measures to improve HDP care.

### Data analysis

Data analysis followed a thematic content analysis approach which was done manually. Manual coding was chosen to foster deep immersion in the raw localized narratives, enabling the analytical team to engage in close, iterative transcript reading and collaborative consensus meetings without the abstraction that may sometimes be introduced by the automated software management tools. Transcription was performed verbatim. To ensure analytical rigor, a multi-step coding process was employed. The steps taken include; familiarization: Two reviewers (PM and GM) independently read and re-read a subset of four transcripts. Then we proceeded to have initial coding done. The two researchers independently generated initial codes from this subset. Following this was codebook development. Through consensus meetings involving HML and JN, the team compared independent codes, resolved discrepancies, and developed a preliminary codebook. Application and refinement was the next step. The developed codebook was applied to the remaining transcripts using an inductive framework, allowing new codes to emerge iteratively. Thematic Synthesis was finally done where related codes were grouped into the four broad themes.

Although member checking was not performed due to logistical constraints, the final themes were reviewed by the entire research team to ensure they were strictly grounded in the participants’ original narratives to ensure accuracy and credibility of the results.

## Results

We conducted 13 interviews during the study period and the characteristics of the study participants are shown in Table 1. The majority of the participants were aged 30–40 years, female, midwives, and held a diploma in health sciences.

**Table 1:** Showing participant characteristics

Characteristic *n* = 13	Category	*n* (%)
Age	20–29	4 (30.8)
	30–40	5 (38.4)
	> 40	4 (30.8)
Sex	Female	9 (69.2)
	Male	4 (30.8)
Cadre	Clinical officer	3 (23.1)
	Midwife	8 (61.5)
	Nurse	1 (7.7)
	Medical officer	1 (7.7)
Qualifications	Certificate	5 (38.4)
	Diploma	7 (53.9)
	Degree	1 (7.7)

## Themes generated from the study

We identified four themes from the interviews: (1) Current clinical practices in HDP care, (2) Health system and contextual barriers (3) Key facilitators of primary HDP care, and (4) Provider proposed measures for system improvement as shown in Table 2.

**Table 2:** Showing the study themes and sub-themes

Themes	Sub-themes
Current clinical practices in HDP care	Clinical screening and diagnostic procedures
	Risk-factor-based screening
	Initial stabilization using Magnesium Sulfate and anti-hypertensives
	Health education, psychosocial support, and preventive measures
	Pre-referral care, documentation, and referral pathways
Health system and contextual barriers	Inadequate tools and guidelines for managing HDP
	Equipment and supply constraints
	Understaffing and heavy workload
	Negative cultural beliefs and low socioeconomic status
Key facilitators of primary HDP care	Digital consultation and coordination systems
	Strengthening routine health education
Provider proposed measures for system improvement	Capacity building through continuous clinical mentorship
	Strengthening the supply chain and human resource allocation
	Community-led engagement for early ANC enrolment
	Leveraging technological interventions for decision support
	Strategic redesign of documentation and risk-tracking tools

### Theme 1: Current clinical practices in HDP care

#### Clinical screening and diagnostic procedures

HCPs at primary care facilities primarily rely on a triad of clinical assessments, namely measurement of blood pressure, urinalysis for proteinuria, and physical examination for edema. Screening is typically integrated into the routine ANC workflow, beginning with health education followed by vital sign checks.

*When women come in… we start the health education talk… Then after we take their blood pressure and weight. That is when we rule out those with or without pre-eclampsia.* (Registered Midwife, HCIII).

For cases where elevated blood pressure (≥ 140/90 mmHg) was detected, the diagnostic process involved immediate referral for higher-level clinical review and more extensive laboratory investigations to classify the severity of the disorder.

*Whenever we find that there is a mother with high blood pressure… we refer them for a Medical Officer’s review in the high-risk clinic. We do several tests on them—urinalysis*,* complete blood count*,* renal and liver function tests—to classify their preeclampsia.* (Medical Officer, HCIV).

#### Risk-factor-based screening

A significant component of the screening process also involved identifying clinical and social risk factors during the initial history-taking. HCPs reported assessing for a family history of hypertension, pre-existing chronic conditions like diabetes, advanced maternal age (> 35 years), and obstetric history such as prior HDP episodes.

*We usually ask them whether they have a mother*,* first-degree relative*,* who has ever suffered from hypertension or any hypertensive disorder.* (Enrolled Nurse, HCIII).

#### Initial stabilization using Magnesium Sulfate and anti-hypertensives

Once a diagnosis of HDP was made, the immediate priority for HCPs was clinical stabilization to prevent complications such as eclampsia. The practice was standardized around the administration of loading doses of Magnesium Sulfate and antihypertensive medications before the patient is moved to a higher level of care.

*We give her a loading dose of magnesium sulfate. Then we give her antihypertensives*,* after we refer her for further examinations and investigations and other things we don’t do here.* (Registered Midwife, HCIII).

#### Health education, psychosocial support, and preventive measures

When asked about pharmacological and non-pharmacological preventive strategies used during routine ANC, healthcare providers highlighted dietary counseling and calcium supplementation (if available at the facility). Notably, despite targeted probing on prophylaxis for high risk mothers, no healthcare provider (0/13) mentioned the routine prescription or use of prophylactic low-dose aspirin. HCP also provided psychosocial support.

*I reassure the patient*,* counsel the patient because when a woman is at a lower health center and you tell her to go to Mbarara [Regional Referral Hospital]*,* she panics a lot and the blood pressure may even go higher so you counsel the patient.* (Registered Midwife, HCIII).

*We counsel the mother*,* and we advise on diet… reduce salt intake*,* sugar intake*,* and take a lot of water… Sometimes we always give calcium supplements or sometimes when we don’t have… we write for that person to be taking while they come to check weekly.* (Midwife/Nurse, HCIII).

#### Pre-referral care, documentation, and referral pathways

The final stage of HDP management involved a coordinated referral process. This included the physical preparation, such as inserting a cannula and arranging transport, alongside rigorous documentation on ANC cards or exercise books. Communication with receiving facilities was increasingly facilitated through digital platforms, ensuring a continuity of care during the transition.

*We give her a loading dose of magnesium sulfate. Of course*,* we insert a cannula… and send them. Another thing you escort her and also go with more magnesium in case she gets an episode along the way.* (Registered Midwife, HCIII).

*We always make sure that we document everything on the antenatal card… Or when we have sent her to the referral there is a system of Ankore LMNS [WhatsApp group] they keep updating on the group how the woman that has been referred is doing.* (Midwife, HCIII).

### Theme 2: Health system and contextual barriers

#### Inadequate tools and guidelines for managing HDP

Participants indicated that while clinical knowledge exists, there was a lack of specialized, accessible tools such as HDP-specific checklists or localized job aids. Instead of following standardized protocols at the bedside, HCPs often relied on their foundational medical training or general textbooks, which may not offer the rapid-reference support needed during busy clinics.

*We don’t have checklists or guides when screening for the risk factos of HDP. We follow what we studied at school*,* we also have a book that we read and get to know how to manage them.* (Midwife, HCIII).

#### Equipment and supply constraints

HCPs highlighted barriers such as malfunctioning blood pressure machines due to lack of batteries and intermittent stock-outs of essential consumables like urine dipsticks. These technical failures introduced diagnostic uncertainty and delayed the classification of pre-eclampsia.

*We find it difficult because sometimes you find that the BP machine has no batteries*,* that can make you get errors. Another challenge we have is laboratory. You’re suspecting to be preeclamptic*,* but when you go to laboratory*,* you don’t have a laboratory assistant.* (Midwife, HCIII).

Sometimes the urine dipsticks are out of stock. Enrolled Nurse, HCIII.

#### **Understaffing and heavy workload**

Participants described understaffing as a common issue that creates an unconducive working environment because it may lead to compromised clinical precision. They assert that heavy workload often led to the delegation of triage duties to less-trained personnel, such as Village Health Teams (VHTs), or resulted in essential steps such as blood pressure monitoring being inadvertently skipped as staff rotated between different clinical departments.

*Due to under staffing… on triage*,* you are forced to put there the VHT to cover. You find that there is like VHT yet he or she cannot interpret the blood pressure reading.* (Clinical Officer, HCIII).

*We are only two health workers. Therefore*,* by the time a midwife comes to the antenatal clinic after completing duties in the other departments*,* she might even forget to take blood pressure for some women.* (Midwife, HCIII).

#### Negative cultural beliefs and socioeconomic status

Providers identified that patient-level factors, specifically cultural interpretations of HDP symptoms, acted as a barrier to timely care. Some patients attributed symptoms of hypertension to “witchcraft,” which resulted in the rejection of clinical explanations and findings. In addition, patients’ inability to afford private laboratory tests or transportation constrained care delivery and limited healthcare providers’ ability to act on clinical findings.

*They have their own beliefs like someone is bewitching them… when you tell her that she is hypertensive*,* she shows you that she doesn’t have it.* (Nurse/Midwife, HCIII).

*When a woman has no money to carry out the laboratory tests*,* our hands are tied. We do not have an option. You find that causing problems.”*(Midwife, HCIII).

### Theme 3: Key facilitators of primary HDP care

#### Digital consultation and coordination systems

The use of digital platforms transformed how primary care workers managed high-risk pregnancies. Real-time digital consultation through platforms like the *Ankore local maternity and neonatal service (LMNS)*, a WhatsApp group, enabled immediate peer support and access to specialist advice, effectively bridging the gap between primary health centers and tertiary-level facilities.

*There is a system of Ankore LMNS they keep updating on the WhatsApp group… The benefit is that any alarm you make; you cannot fail to be helped. I use my phone to call a doctor… or I post that case on Ankole LMNS. Yes I hear back*,* immediately.* (Registered Midwife, HCIII).

#### Strengthening routine health education

HCPs emphasized that routine health education sessions were a powerful facilitator for screening. Through consistent education of mothers on nutrition, stress reduction, and the importance of regular follow-ups, providers fostered an environment of awareness that encouraged timely clinical visits and improved adherence to diagnostic procedures.

*We always counsel the women about nutrition. We educate on diet and*,* sometimes ask them to always come back weekly to check their blood pressure.* (Medical Officer, HCIV).

### Theme 4: Provider proposed measures for system improvement

HCPs proposed a series of strategic measures aimed at strengthening the clinical and social framework of HDP care. By reflecting on barriers in their daily workflows—such as equipment failure, high workload, and late patient presentation—participants identified solutions that emphasized professional accountability, task-sharing, technology support and improved referral coordination.

#### Capacity building through continuous clinical mentorship

Participants stated that one-off training was insufficient and proposed ongoing, focused capacity building where experienced specialists provideds facility-based mentorships. They believed this approach would bridge the gap between their foundational training and the evolving complexities of HDP management.

*If possible*,* people with experience*,* they can always come around*,* teach us the staff of these centres. They can also always put up conferences for us to attend where we can be equipped with such knowledge…* (Clinical Officer, HCIV).

#### Strengthening the supply chain and human resource allocation

To mitigate the risk of clinical error caused by burnout and equipment failure, HCPs emphasized the need for a stable supply of functional tools and a more robust workforce. They suggested that increasing staffing levels at the triage level was essential to ensure that vital screenings, like blood pressure and urinalysis, were not skipped due to high patient volumes.

*If you can provide us with enough BP machines*,* that can work so that we screen more mothers.* Clinical Officer, HCIII.

*They should employ more workers because we are few workers. You find that you are the one to clerk*,* take the BP and at the same time you are the one to carry out antenatal. Sometimes you can make errors.* Enrolled Nurse, HCIII.

#### Community-led engagement for early ANC enrollment

Recognizing that some barriers originated outside the clinic, participants suggested extending health education into community settings. By engaging mothers through churches, markets, and radio, they perceived a potential to promote early ANC attendance, thereby enabling the identification of HDP risks well before the third trimester.

*Health education talks that is in churches*,* markets*,* radio talk shows. It can help mothers attend antenatal early. Some mothers come during their third trimester. So*,* I wish they could come for antenatal earlier during their early first trimester.* (Clinical Officer, HCIV).

#### Leveraging technological interventions for decision support

Mobile health applications were perceived as a significant measure to address the absence of physical clinical guidelines. HCPs noted that such applications could deliver real-time alerts and decision support, reducing the cognitive burden associated with recalling protocols during high-pressure clinical encounters.

*A mobile app is good. You might find that I have spent a very long time without reading through the guidelines. But when I am taking BP and the app is telling me do this or that*,* it’s guiding me. I welcome it.* (Registered Midwife, HCIII).

#### Strategic redesign of documentation and risk-tracking tools

Participants proposed practical changes to documentation tools, such as the national antenatal card. They suggested prioritizing the clear visual display of risk factors to ensure that high-risk cases were immediately identifiable to all providers involved in the patient care pathway.

*I would suggest that those risk factors come on the first page so that when you are referred*,* the risk factors can easily be seen. They should make the information on risk factos more visible.* (Registered Midwife, HCIII).

## Discussion

This study explored the perspectives of HCPs on the screening, diagnosis, and management of HDP in southwestern Uganda. Our findings reveal a resilient but strained primary healthcare system where clinical practices are frequently shaped by resource scarcity, necessitating a reliance on individual discretion and informal digital innovations to bridge the gap between national guidelines and bedside reality.

Although our study adopted an inductive approach to capture participant perspectives, our findings resonate with the WHO Health Systems Framework [11]. The themes that emerged map directly across key building blocks of health systems; service delivery and medical technologies were constrained by non-functional blood pressure machines and urine dipstick stock-outs, health workforce challenges were evident in understaffing and heavy workloads, and health information systems were adapted through informal mHealth channels (WhatsApp) and suggested mHealth decision-support tools. The health systems framework shows that HCP knowledge alone cannot overcome structural, multi-block vulnerabilities without targeted systems-level strengthening.

HCPs highlighted that the screening for HDP relied on the triad of blood pressure measurement, proteinuria assessment, and risk-factor screening. However, our results show that while the clinical intent is present, the functional capacity is often absent. The reliance on non-functional equipment—such as BP machines lacking batteries—and intermittent stock-outs of urine dipsticks or lack of laboratories introduces diagnostic uncertainty. These findings align with global literature on challenges in managing HDP in low-resource settings and such challenges have also been shown to affect the quality of care received by women with HDP [12, 13].

The complete absence of low-dose aspirin as a reported prophylactic measure among frontline providers represents a critical missed opportunity for HDP prevention. While international guidelines, such as those from the International Society for the Study of Hypertension in Pregnancy, advocate for early prophylactic intervention [14], our findings suggest that without standardized, simple screening algorithms at primary level health facilities, providers struggle to identify the high-risk cohort eligible for such treatment. Studies have shown that the limited use of prophylactic aspirin may be due to lack of standardized risk screening algorithms, low provider knowledge, late initiation of ANC attendance (missing the crucial therapeutic window below 16 weeks of gestation) and supply chain vulnerabilities leading to stock-outs of low dose aspirin at the primary level [15–17]. Strengthening the capacity of primary-level facilities to initiate prophylaxis could significantly reduce the burden of severe preeclampsia at tertiary referral facilities.

A notable and somewhat unexpected facilitator identified in our study was the informal yet highly effective use of WhatsApp—through the *Ankole Local Maternity and Neonatal Service (LMNS)* group—for clinical consultation. While mobile health (mHealth) interventions are often discussed in the literature as formal, purpose-built applications and have been shown to improve maternal health is sub-saharan Africa [18, 19], our participants have repurposed a social media tool to bridge the gap between primary and tertiary care levels. This digital adoption demonstrates HCP readiness for mHealth-led decision support systems as has been described elsewhere in Ghana [20]. Despite its operational utility, relying on WhatsApp for clinical decision support may introduce data privacy, ethical and medico-legal risks. Sharing patient information, images or clinical information over such platforms may risk violation of the local data protection regulations, particularly in absence of standardized patient consent processes [21–23]. These platforms may also lack formal governance around clinical liability if an adverse outcome occurs following peer advice given over WhatsApp.

Although the use of WhatsApp presents privacy and regulatory challenges, its widespread use presents a unique opportunity for scalable and sustainable mHealth intergration. However, when compared to purpose-built, formal mHealth decision support applications which offer standardized triage algorithms, offline capability, automated audit trails and integrated electronic medical records, WhatsApp may serve as a temporary bridge rather than a permanent solution [24, 25]. WhatsApp leverages existing digital literacy, low-bandwidth optimization and personally accessible hardware, achieving operational scale.

Therefore, as the use of WhatsApp transitions from informal to formal, future research will need to address data privacy and clinical liability concerns associated with using social media platforms for patient-identifiable consultations.

The management of HDP is not merely a clinical challenge but a socio-cultural one. The persistent belief in “witchcraft” as a cause for hypertensive symptoms directly undermines clinical compliance, as evidenced by patients stopping medication once symptoms marginally improve and this finding is similar to other studies in sub-saharan [26]. This underscores the fact that clinical excellence at the health facility level must be complemented by community-led engagement. Participants’ recommendation to extend health education through churches, markets, and radio talk shows represents a strategic and contextually relevant approach.By promoting early ANC attendance and demystifying HDP symptoms beyond the clinic, the health system can shift from reactive stabilization to proactive, first-trimester screening.

The mitigation measures proposed by healthcare providers, particularly the call for mHealth decision-support tools, represent an important next step. An integrated mobile application could function as a digital mentor, providing real-time guideline reminders and reducing the cognitive load on staff who often face heavy workloads. Such a tool could standardize pre-referral care, ensuring that every woman receives a loading dose of magnesium sulfate regardless of which provider is on duty. In addition, the suggested redesign of antenatal care cards to make risk factors bold and highly visible represents a low-cost, high-impact documentation intervention with the potential to improve continuity of care during referrals.

### Strengths and limitations

A key strength of this study is the diversity of HCP perspectives captured across different primary health facility tiers (HCIII and HCIV), providing a comprehensive view of the primary care landscape. However, the study was limited to Mbarara City, and while these facilities serve a vast catchment area, the findings particularly regarding digital health adoption (WhatsApp consultations) and referral practices may not fully capture the experiences of HCPs in more remote, hard-to-reach rural districts. Our sample included only one medical officer, with the majority being midwives. Although this reflects the natural health workforce composition at the primary care level where midwives are responsible for ANC screening and triage, it may underrepresent higher level diagnostic, management and complex referral decision making perspectives that are typically by medical officers at CEmONC facilities. Additionally, as a qualitative study, the results represent the self-reported practices and perceptions of participants, which may be subject to social desirability bias. Healthcare providers may have felt inclined to portray their routine screening and emergeny management practices in a way that conforms with the expected professional norms. This study also lacked observational validation and therefore it was difficult to triangulate the stated practices actual practice in the facilities. Future research should utilize observational methods to validate clinical practices as they occur within the facilities.

## Conclusions and recommendations

HCPs in southwestern Uganda demonstrated high clinical awareness of HDP care but frequently had to improvise due to inadequate tools, malfunctioning equipment, and understaffing. Their innovative use of digital consultation via WhatsApp, alongside proactive suggestions for community-based health education, reflects a workforce prepared for systemic improvements. Addressing the identified barriers through stabilized supply chains, facility-based mentorship, and the integration of mHealth decision-support tools could shift primary care facilities from being primarily sites of stabilization and referral to functioning as robust hubs for HDP prevention and early diagnosis.

## Supplementary Information

Below is the link to the electronic supplementary material.

Supplementary Material 1

## Data Availability

Data are available upon reasonable request from the corresponding author.

## Abbreviations

ANC: Antenatal Care
Bemoc: Basic Emergency Obstetric Care
BP: Blood Pressure
CemoC: Comprehensive Emergency Obstetric Care
HCP: Healthcare providers
HC: Health Center
HDP: Hypertensive Disorders of Pregnancy
LMNS: Local Maternity and Neonatal Service
MRRH: Mbarara Regional Referral Hospital
MUST: Mbarara University of Science and Technology
UNCST: Uganda National Council for Science and Technology
