# Supplementary material for: Primary healthcare provider perspectives on hypertensive disorders of pregnancy in southwestern Uganda: a qualitative study
Source: BMC Health Serv Res. 2026 Aug 6;26:1524. doi: 10.1186/s12913-026-15315-2 (PMC13733166; doi:10.1186/s12913-026-15315-2)
Supplement: Supplementary file 1 [file 12913_2026_15315_MOESM1_ESM.docx]

**Interview guide for health workers on improving screening and diagnosis of hypertensive disorders of pregnancy**

Demographics

i) Age………………
ii) Sex………………
iii) Cadre (Doctor, Midwife, Nurse, Clinical officer) …………………….
iv) Education level: - Masters, Degree, Diploma, Certificate………………….
1. What is the Health workers ‘current practice on screening, diagnosis of hypertensive disorders of pregnancy and prophylaxis interventions?

- What tools are you currently using to assess a woman’s risk for preeclampsia? (prompts when do you screen? At what gestational age? What are they using to screen? Do they use any biomarkers radiology or proteins, Any checklist used for screening)
- How do health workers make the diagnosis of hypertensive disorders of pregnancy? (Prompts: BP machines, laboratory services, urine protein kits, once the diagnosis is made, what is the clinical management pathway for the clients, what job aids do you have available in case you need to consult to make a decision)
- What are some of the techniques that you use to ensure accurate blood pressure measurements among pregnant women?
- After screening high risk pregnant women, what are some of the prophylactic interventions you are currently giving?

2. What are the challenges in screening and diagnosis of hypertensive disorders of pregnancy and prophylaxis? (prompts: use of guidelines, knowledge of health worker, diagnostic tools)
3. What are Health care workers’ self-reported solutions to challenges in screening and diagnosis of hypertensive disorders of pregnancy and prophylaxis?
4. How best can a screening technique with patient factors (prior preeclampsia, chronic hypertension, pregestational diabetes mellitus, maternal BMI >30kg/m^2^, antiphospholipid syndrome, and assisted reproduction, multiple gestation, advanced age, nulliparity, pregnancy interval > 10 years) be implemented? (Prompt: use of an app? Addition to a woman’s ANC card?)
